# 

**Published:** 1932

**Authors:** 


### The Bristol Medico-Chirurgical Journal

A JOURNAL OF THE MEDICAL SCIENCES FOR THE
WEST OF ENGLAND AND SOUTH WALES.

PUBLISHED UNDER THE AUSPICES OF
*THE BRISTOL MEDICO-CHIRURGICAL SOCIETY.*

EDITOR:
J. A. NIXON, C.M.G., M.D., F.R.C.P.

WITH WHOM ARE ASSOCIATED
E. W. HEY GROVES, M.S., F.R.C.S., B.Sc.
E WATSON-WILLIAMS, M.C., Ch.M., F.R.C.S.
A. E. ILES, O.B.E., M.B., B.S., F.R.C.S.
CAREY F. COOMBS, M.D., F.R.C.P., Assistant Editor.

EDITORIAL SECRETARY:
A. L. FLEMMING, M.B., Ch.B.

---

"Scire est nescire, nisi id me

Scire alius sciret."

---

*VOL. XLIX.*

BRISTOL: J. W. ARROWSMITH LTD.
London: J. W. Arrowsmith (London) Ltd.

R31
.B8

16 2-0486 110 D

### Contents of Vol. XLIX

| | Page |
|---|---|
| Some Observations on the Relationship between Structure and Function. By J. R. Charles, M.A., M.D., F.R.C.P. | 1 |
| Human Improvability. By Charles S. Myers, C.B.E., F.R.S., M.A., M.D., D.Sc. | 31 |
| Trigeminal Neuralgia. By H. H. Carleton, M.D., M.R.C.P. | 47 |
| Some Observations on the Relative Efficacy of the Various Treatments of Pernicious Anæmia. By G. L. Ormerod, M.A., M.B., B.Chir. | 61 |
| Suprarenal Hypertrophy. By J. R. Charles, M.D., F.R.C.P. | 115 |
| Suprarenal Virilism. By J. Antony Birrell, M.D., M.R.C.P. | 119 |
| The Management of the Ear, Nose and Throat in Influenza. By A. J. Wright, M.B., F.R.C.S. | 123 |
| Notes on the Treatment of Some Common Ocular Affections. By E. R. Chambers, F.R.C.S., D.O.M.S. | 131 |
| Notes on Surgical Jaundice. By C. A. Moore, M.S., Ch.M., F.R.C.S. | 139 |
| Insomnia. By R. G. Gordon, M.D., D.Sc., F.R.C.P.E. | 147 |
| Mental Deficiency: An Analysis of the Mental, Physical and Medical Characteristics of a Group of One Hundred and Sixty-Two Adult Feeble-Minded Women. By Richard J. A. Berry, M.D., F.R.C.S., F.R.S.E. | 177 |
| Medical Education. By C. E. S. Flemming, M.R.C.S., L.R.C.P. | 199 |
| Dysphagia with Anæmia, with Reports on Five Cases. By E. Watson-Williams, M.C., Ch.M., F.R.C.S. | 209 |
| Albuminuria in Pregnancy. By R. S. S. Statham, M.D., Ch.M. | 219 |
| Albuminuria in Pregnancy. By J. A. Nixon, C.M.G., M.D., F.R.C.P. | 229 |
| Notes on Female Circumcision as Practised by the Ameru. By H. W. Brassington, M.B., Ch.B. | 237 |
| The British Pharmacopœia, 1932. By A. L. Taylor, M.P.S. | 241 |
| The Long Fox Memorial Lecture: The Problem of Deafness. By A. J. M. Wright, M.B., B.S. Lond., M.B., Ch.B. Brist., F.R.C.S. | 255 |
| Prognosis in Coronary Thrombosis. By Carey F. Coombs, M.D., F.R.C.P. | 277 |
| The Effects of Lightning: with Especial Reference to the Nervous System. By Macdonald Critchley, M.D., F.R.C.P. | 285 |
| The Care and Management of Premature Infants. By Francis J. Hector, M.D., F.R.C.S. | 301 |
| Note on a Case of Ponto-Cerebellar Tumour in a Girl of Six Years. By A. Wilfrid Adams, M.S., F.R.C.S. | 309 |
| Provincial Medical Journals. By J. A. Nixon, C.M.G., M.D., F.R.C.P. | 311 |
| Reviews of Books | 87, 157, 246, 319 |
| Editorial Notes | 95, 166, 250, 325 |
| Obituaries | 251, 252, 326 |
| Meetings of Societies | 99, 170, 329 |
| The Medical Library of the University of Bristol | 103, 172, 253, 332 |
| Local Medical Notes | 105, 174, 254, 333 |

### The Medical Library of the University of Bristol,

WITH WHICH IS INCORPORATED

### The Library of the Bristol Medico-Chirurgical Society.

---

*The following donations have been received since the publication of the list in December,* 1931.

March, 1932.

| | |
|---|---|
| A. G. Timbrell Fisher, Esq., M.C., F.R.C.S. (1) | 1 volume. |
| Cecil A. Joll, Esq., M.S., B.Sc., F.R.C.S. (2).. | 1 ,, |
| London School of Hygiene and Tropical Medicine (3) .. .. .. .. .. | 1 ,, |
| Dr. M. Nierenstein (4) .. .. .. .. | 1 ,, |
| Registrar of the University of Bristol (5) .. | 1 ,, |
| Royal College of Surgeons of England (6) .. | 1 ,, |
| Smithsonian Institution (7) .. .. .. | 1 ,, |
| Cyril H. Walker, Esq., B.A., M.B., F.R.C.S. (8) | 39 volumes. |

Unbound periodicals have also been received from Professor E. W. Hey Groves and Dr. Carey F. Coombs.

### THE ONE HUNDRED AND THIRTY-NINTH LIST OF BOOKS.

The figures in round brackets refer to the figures after the names of the donors. The books to which no such figures are attached have either been bought from the Library Fund or received through the *Journal.*

**Beaumont, W.** .. .. *Fundamental Principles of Ray Therapy* .. 1931
**Bellingham-Smith, E. and Feiling, A.** *Modern Medical Treatment.* 2 vols. 1931
**Brockbank, E. M.** .. *Conduct of Life Assurance Examinations* .. 1931
**Burdon-Cooper, J., and Roberts, A.** *Studies in the Photo-Activity and Therapy of the Tungsten-Titanium Arc* .. 1931
**Colwell, H. A.** .. .. *Notes on Radium Therapy for Medical Students* .. .. .. .. .. .. .. .. 1931
**Darwin, E.**.. .. .. *Zoönomia, or the Laws of Organic Life* 2 Vols. (8) 1796
**Delmege, J. A.** .. .. *Towards National Health* .. .. .. .. 1931
**Donaldson, R.** .. .. *Practical Morbid Histology* .. .. .. .. 1931
**Drewitt, F. D.** .. .. *Life of Edward Jenner* .. .. .. .. .. .. 1931
**Fisher, A. G. T.** .. *Internal Derangements of the Knee Joint* (1) 1924
**Gould, G. M.** .. .. *Gould's Medical Dictionary* .. .. 3rd Ed. 1931
**Handley, W. S.** .. *Genesis of Cancer* .. .. .. .. .. .. .. 1931
**Johnson, G. L.** .. .. *Pocket Atlas and Textbook of the Fundus Oculi* 1931
**Joll, C. A.** .. .. *Diseases of the Thyroid Gland* .. .. (2) 1932
**McDowall, R. J. S.** .. *Science and Signs of Symptoms* .. .. .. 1931
**Martini, J., and Joselevich, M.** *La. Estenosis Dextroventriculas*.. .. 1931
**Nott, H. W.** .. .. *Thyroid and Manganese Treatment* .. .. 1931
**Scott, S. G.** .. .. *Radiology in Relation to Medical Jurisprudence* 1931
**Souttar, H. S.** .. .. *Radium and Cancer* .. .. .. .. .. .. 1932
**Stokes, J. H.** .. .. *Modern Clinical Syphilology* .. .. .. .. 1928
**Sykes, W. S.** .. .. *Manual of General Medical Practice* 2nd Ed. 1931
**Travers, M. W.**.. .. *Discovery of the Rare Gases* .. .. .. (8) 1928
**Van de Velde, T. H.** *Sex Hostility in Marriage* .. .. .. .. 1931
**Van de Velde, T. H.** *Fertility and Sterility in Marriage* .. .. 1931
**Vickery, H. B., and Schmidt, C. L. A.** *History and Discovery of the Amino Acids* .. .. .. .. .. .. (4)
**Warwick and Tunstall** *First-aid to the Injured and Sick* 13th Ed. 1931
**White, P. D.** .. .. *Heart Disease* .. .. .. .. .. .. .. .. 1931

### TRANSACTIONS, REPORTS, JOURNALS, ETC.

Association of American Physicians, Transactions. Vol. XLVI. .. 1931
English Catalogue of Books, 1931 .. .. .. .. .. .. .. .. .. .. 1932
Kelly's Directory of Bristol and Suburbs .. .. .. .. .. .. .. 1932
London School of Hygiene and Tropical Medicine, Collected Addresses and Laboratory Studies. VII. .. .. .. .. .. .. .. (3) 1930–31
Ophthalmic Review. Vol. X.—XXXV. .. .. .. .. .. (8) 1891–1916
Ophthalmological Society Transactions. Vol. LI. .. .. .. .. .. 1932
Progressive Medicine. Vol. 4, December .. .. .. .. .. .. .. 1931
Quarterly Cumulative Index Medicus. IX. .. .. .. .. .. .. .. 1931
Royal College of Surgeons of England, a list of the Transactions, Periodicals and Memorandums in the Library. 2nd Ed. .. (6) 1931

| | | |
|---|---|---|
| Saint Bartholomew's Hospital Reports. Vol. LXIV. .. .. .. .. | | 1931 |
| Smithsonian Institution. Annual Report, 1930 .. .. .. .. .. | (7) | 1930 |
| Surgical Clinics of North America. Vol. 11, No. 6, December .. | | 1931 |
| Surgical Clinics of North America. Vol. 12, No. 1, February .. | | 1932 |
| Whitaker's Almanack .. .. .. .. .. .. .. .. .. .. .. | (5) | 1932 |

### Local Medical Notes

EXAMINATION RESULTS.

**University of Bristol.**—Students of the University have recently passed the following examinations :—

M.B., Ch.B. (*Part I.*).—*Second Examination. Pass :* R. D. Cæsar, Diana R. Eyre-Brook, Monica L. Hawkins, Ursula G. Hewitt, E. J. Lace, J. S. W. Little, N. R. Matheson, Coralie W. Rendle-Short, L. A. Schnipelsky, J. W. E. Snawdon, E. L. Ward, P. Zimmering. *In Organic Chemistry* (*Completing Examination*) *:* D. S. Pritchard. *In Elementary Anatomy* (*Completing Examination*) *:* G. W. Vowles, Ursula W. Wood. *In Elementary Anatomy only :* Margaret E. Morgan.

M.B., Ch.B. (*Part II.*). *Second Examination. Pass :* V. T. Baxter, Kathleen G. Brimelow, F. C. Collingwood, Theodora M. Crabtree, J. G. Field, P. N. Heron, A. E. Jowett, S. D. Loxton, M. A. Nicholson, B. Ridgway (with distinction in Anatomy), A. W. Woolley.

L.D.S.—*First Professional Examination. Pass : In Dental Anatomy* (*Completing Examination*) *:* J. G. Clancy. *In Dental Anatomy* (*Completing Requirements*) *:* J. Hewitt. *In Dental Anatomy only :* Frances M. Orton, L. E. Scull.

**Conjoint Board** — M.R.C.S., L.R.C.P. — *In Chemistry :* Myrtle M. Baxter. *In Elementary Biology :* J. T. S. Hutchins. *In Medicine :* A. H. Bulleid,* W. Woolley,* W. L. Sleight,* N. C. Coombs. *In Medicine and Surgery :* J. J. Kempton.* *In Midwifery :* D. P. Dewe, W. R. Cambridge. *In Surgery :* A. M. Davies, G. M. Evans.

L.D.S., R.C.S.—*First Professional Examination. Part I.* (*Dental Mechanics*) *and Part III.* (*b*) (*Dental Anatomy*) *:*

* Qualified.

B. H. W. Baker. *Part I.* (*Dental Mechanics*) *:* Margaret D. Hooper, L. Jonathan, N. E. Miles. *Part II.* (*Dental Metallurgy*) *:* T. H. Williams. *Dental Anatomy :* C. A. G. Evans.

---

APPOINTMENTS.

R. R. Garden, M.A., M.B., Ch.B. (Aberd.), D.P.H., D.O.M.S., to be Assistant Surgeon to the Bristol Eye Hospital.

N. H. Kettlewell, H.D.D., R.C.S. (Edin.), L.D.S., R.C.S. (Eng.), L.M.S.S.A. (Lond.), to be Assistant Lecturer in Orthodontics in the University of Bristol.

---

**University of Liverpool.**—The Milne Medal in Tropical Medicine has been awarded to H. J. H. Spreadbury, M.B., Ch.B. Bristol.

### Bristol Medico-Chirurgical Society.

### President.

J. R. CHARLES, M.A., M.D. Cantab., F.R.C.P.

### President-Elect.

E. W. HEY GROVES, M.S., F.R.C.S., B.Sc. Lond.

### Committee.

*Ex-Officio* { J. A. NIXON, *C.M.G.*, B.A., M.D. Cantab., F.R.C.P. (Editor of *Journal*).
G. PARKER, M.A., M.D. Cantab. (Hon. Librarian).

*Elected.*

W. K. WILLS, *O.B.E.*, M.A., M.B., B.Ch. Cantab. (Ex-President).
DUNCAN WOOD, F.R.C.S.
H. CHITTY, M.S. Lond., F.R.C.S.
A. R. SHORT, B.Sc., M.D. Lond., F.R.C.S.
H. G. KYLE, M.A., M.D., B.Ch. Oxon.
W. P. KENNEDY, M.D. Dub.
CAREY F. COOMBS, M.D., F.R.C.P.
W. A. JACKMAN, M.B., F.R.C.S. Ed.
H. ELWIN HARRIS, Jun., M.A., M.B. Cantab., F.R.C.S.

### Honorary Secretary.

H. L. SHEPHERD, M.B., Ch.M. Brist.

### Honorary Treasurer.

A. E. ILES, *O.B.E.*, M.B., B.S. Lond., F.R.C.S.

### University Library Sub-Committee.

G. PARKER, M.A., M.D. Cantab. A. R. SHORT, B.Sc., M.D., B.S. Lond., F.R.C.S. J. O. SYMES, M.D. Lond.

Medical Practitioners wishing to join the Society are requested to communicate with the Hon. Secretary, Mr. H. L. Shepherd, 16 Victoria Square, Clifton. The Annual Subscription is £1 11s. 6d., payable to Hon. Treasurer.

---

*Extracts from Journal By-laws.*

4.—The *Journal* shall be delivered free to Subscribers and also to Members of the Society whose subscriptions are not in arrear.

6.—The Annual Subscription to the *Journal* is 10/6, payable to Hon. Treasurer.

---

Communications intended for insertion in the *Journal* should be sent to the Editor, Dr. J. A. Nixon, 7 Lansdown Place, Clifton, Bristol.

Books for Review and Exchange Journals should be sent to the Assistant-Editor, *Bristol Medico-Chirurgical Journal*, Medical Library, The University, Bristol.

Communications referring to the delivery of the *Journal* should be sent to the Editorial Secretary, A. L. Flemming, 48 Pembroke Road, Clifton, Bristol.

Cases for Binding Vols. I—XLVIII are now ready, and may be obtained price 1/9 each (postage extra), upon application to J. W. Arrowsmith Ltd., Quay Street, Bristol; or the *Journal* will be bound, including Case, for 7/- per volume.

### Bristol Medico-Chirurgical Society.

### PAST PRESIDENTS.

1874—75. FREDERICK BRITTAN, M.D., B.A. Dub.
1875—76. ROBERT WILLIAM COE.
1876—77. SAMUEL MARTYN, M.D. Ed.
1877—78. AUGUSTIN PRICHARD, M.D. Berlin.
1878—79. HENRY EDWARD FRIPP, M.D. Würzburg.
1879—80. WILLIAM MICHELL CLARKE.
1880—81. JOSEPH GRIFFITHS SWAYNE, M.D. Lond.
1881—82. EDWARD LONG FOX, M.D. Oxon.
1882—83. JAMES GEORGE DAVEY, M.D. St. And.
1883—84. WILLIAM JOHNSTONE FYFFE, M.D., B.A. Dub.
1884—85. GEORGE FORSTER BURDER, M.D. Aberd.
1885—86. WILLIAM HENRY SPENCER, M.D., M.A. Cantab.
1886—87. FRANCIS POOLE LANSDOWN.
1887—88. LEMUEL MATTHEWS GRIFFITHS.
1888—89. ROBERT SHINGLETON SMITH, M.D., B.Sc. Lond.
1889—90. NELSON CONGREVE DOBSON, Ch.M. Brist.
1890—91. SAMUEL HENRY SWAYNE.
1891—92. FRANCIS RICHARDSON CROSS, M.B. Lond., LL.D. Brist.
1892—93. EDWARD MARKHAM SKERRITT, M.D., B.S., B.A. Lond.
1893—94. JAMES GREIG SMITH, M.B., C.M., M.A. Aberd., F.R.S.E.
1894—95. ALFRED JAMES HARRISON, M.B. Lond.
1895—96. ARTHUR WILLIAM PRICHARD.
1896—97. ALFRED EDWARD AUST LAWRENCE, M.D., C.M. Aberd.
1897—98. JOHN EDWARD SHAW, M.B., C.M. Ed.
1898—99. ROBERT ROXBURGH, M.B. Ed.
1899—00. WILLIAM HENRY HARSANT.
1900—01. DAVID SAMUEL DAVIES, M.D. Lond.
1901—02. BARCLAY JOSIAH BARON, M.B., C.M. Ed.
1902—03. GEORGE MUNRO SMITH, M.D. Brist.
1903—04. JAMES PAUL BUSH, C.M.G., C.B.E., Ch.M. Brist.
1904—05. REGINALD EAGER, M.D. Lond.
1905—06. JOHN DACRE.
1906—07. JAMES TAYLOR.
1907—08. HENRY WALDO, M.D., C.M. Aberd.
1908—09. JOHN MICHELL CLARKE, M.D , M.A. Cantab.
1909—10. JAMES SWAIN, C.B., C.B.E., M.D., M.S. Lond.
1910—11. BERTRAM MITFORD HERON ROGERS, M.D., B.Ch., B.A. Oxon.
1911—12. CHARLES ALEXANDER MORTON.
1912—13. WALTER CARLESS SWAYNE, M.D., B.S. Lond. and Brist.
1913—14. PATRICK WATSON-WILLIAMS, M.D. Lond.
1914—15. WILLIAM HARRY CHRISTOPHER NEWNHAM, M A., M.B. Cantab.
1915—16 GEORGE PARKER, M.A., M.D. Cantab.
1916—17 FRANCIS HENRY EDGEWORTH, M.A., M.D. Cantab., D.Sc. Lond.
1917—18 FREDERICK LACE.
1918—19 ROBERT GUTHRIE POOLE LANSDOWN, M.D., B.S. Durh.
1919—20 I. WALKER HALL, M.D., Ch.B. Vict.
1920—21 LEOPOLD ERNEST VALENTINE EVERY-CLAYTON, M.D. Lond.
1921—22 CYRIL HUTCHINSON WALKER, M.B., B.A. Cantab.
1922—23 JOHN ALEXANDER NIXON, C.M.G., B.A., M.D. Cantab.
1923—24 JOHN LACY FIRTH, M.D., M.S Lond.
1924—25 JOHN ODERY SYMES, M.D. Lond.
1925—26 THOMAS CARWARDINE, M.S. Lond.
1926—27 ARTHUR LAUNCELOT FLEMMING, M.B., Ch.B. Brist.
1927—28 WALTER KENNETH WILLS, M.A., M.B., B.Ch. Cantab.
1928—29 HENRY LAWRENCE ORMEROD, M.D., B.Ch. R.U.I.
1929—30 CHARLES FERRIER WALTERS.
1930—31 DAVID CHARLES RAYNER, Ch.M. Brist.

1932.

### LIST OF SUBSCRIBERS

TO THE

### Bristol Medico-Chirurgical Journal.

### HONORARY MEMBERS OF THE SOCIETY.

| | |
|---|---|
| *Swain, James, *C.B.*, *C.B.E.*, M.D., M.S. Lond. .. .. .. .. | Wyndham House, Easton-in-Gordano. |
| *Watson-Williams, P., M.D.Lond. | 2 Rodney Place, Clifton. |
| *Smith, W. A., M.A., M.B. Oxon. | 70 Pembroke Road, Clifton. |

### SUBSCRIBERS.

*Those marked * are Members of the Society.*

| | |
|---|---|
| *Adams, A. W., M.S. Lond. ... ... ... ... ... | Rodney House, Clifton. |
| *Aldwinckle, H. M. M.B., Ch.B. Brist. ... ... | 30 E Berkeley Square, Clifton. |
| *Alexander, D. A., M.B., Ch.B. Vict. ... ... | 112 Pembroke Road, Clifton, Bristol. |
| Alexander, G. L., M.B., B.Ch. Cantab. ... ... | West African Medical Service, Laura, via Accra, Gold Coast. |
| *Alford, A. F., M.B., Ch.B. Brist. ... ... ... | 84 Queens Road, Bristol. |
| Alford, H. J., M.D. Lond. ... ... ... ... ... | 4 Park Terrace, Taunton. |
| *Armstrong, Jean, M.B., Ch.B. Brist. ... ... | Ryecroft, Bell Barn Road, Stoke Bishop, Bristol. |
| *Aubrey, T., M.B. Lond. ... ... ... ... ... | Bitton, near Bristol. |
| *Bailey, Hamilton... ... ... ... ... ... ... | 240 Hendon Way, London, N. |
| *Baker, Lily A., M.D., B.Ch., B.A.O., R.U.I. | Clifton Down House. |
| *Barber, M. C., M.B., Ch.B. Brist. ... ... ... | Ingledene, High Street, Staple Hill, Bristol. |
| *Barker, G. H., M.D., B.Sc. Lond. ... ... ... | 124 Redland Road, Bristol. |
| *Bartholomew, E. U. ... ... ... ... ... ... | 12 Lansdown Place, Clifton. |
| *Bates, R. ... ... ... ... ... ... ... ... | Stoke Park Colony, Bristol. |
| *Bates, Evelyn ... ... ... ... ... ... ... | Stoke Park Colony, Bristol. |
| *Batt, J. C., M.B., Ch.B. Brist. ... ... ... | Maudsley Hospital, London, S.W. |
| Beatson, D. H., M.B., Ch.B. Brist. ... ... ... | 8 Richmond Park Road, Clifton. |
| *Benson, Elizabeth, M.B., Ch.B. Brist. ... ... | 8 Theresa Place, Bristol Road, Gloucester. |
| *Bergin, F. G. ... ... ... ... ... ... ... | 31 Oakfield Road, Clifton. |
| Bernard, C. ... ... ... ... ... ... ... ... | 564 Fishponds Road, Fishponds, Bristol. |
| *Berrill, T. H., M.B., Ch.B. Brist. ... ... ... | Royal Northern Hospital, London, N.7. |
| Berry, F. C., M.D., B.Ch., B.A. Dub. ... ... | Locarno, Burnham, Somerset. |
| *Berry, R. J. A., Prof., M.D. ... ... ... ... | Director of Medical Services, Stoke Park Colony, Bristol. |
| *Binns, R. T., M.B., B.S. ... ... ... ... ... | Combehaven, Hanham. |
| *Birrell, J. A., M.D., M.S. Lond. ... ... ... | 2 Dundonald Road, Redland, Bristol. |
| *Blachford, J. V., *C.B.E.*, M.D. Durh. ... ... | Milverton House, Long Ashton. |
| Blackett, J. F., M.D., B.S. Lond. ... ... ... | Hamsterley, Bloomfield Park, Bath. |
| *Blathwayt, A. de V. ... ... ... ... ... ... | 6 Brock Street, Bath. |
| Bletchly, G. Playne, M.B. Lond. ... ... ... | Hazelwood, Nailsworth, Glos. |
| *Bodman, A. P., M.B., Ch.B. ... ... ... ... | 11 Hurle Crescent, Clifton, Bristol. |
| *Bodman, C. O., M.D. Durh. ... ... ... ... | 17 Upper Belgrave Road, Clifton. |
| *Bodman, F. H., M.B., Ch.B. Brist. ... ... ... | 2 Redland Court Road, Bristol. |
| *Bodman, J. Hervey, M.D. Lond. ... ... ... | 11 Hurle Crescent, Clifton, Bristol. |
| *Boulton, B. J., M.B., Ch.B. Brist. ... ... ... | Ham Green Hospital. |
| *Bradbeer, E. G., M.B., Ch.B. Brist. ... ... | Hillside, Cranbrook Road, Bristol. |
| *Bradley, W. H., B.M., B.Ch. Oxon. ... ... | St. Wulstan's, Stratton-on-Fosse, Bath. |
| *Bray, G., Lt.-Col., *D.S.O.* ... ... ... ... ... | 7 Amherst Road, Ealing, London, W.13 |
| *Brentnall, T. C. ... ... ... ... ... ... ... | Bellfield, Brightlingsea, Essex. |
| *Brierley, C. E. ... ... ... ... ... ... ... | Great Totton, Hants. |

| | |
|---|---|
| *Brinckman, W. P., M.B., Ch.B. Brist. ... ... | 4 Oakland Road, Redland, Bristol. |
| *Bristowe, H. C., M.D. Lond. ... ... ... ... | 3 Upper Belgrave Road, Clifton. |
| *Brittain, H. A., M.B., Ch.B. Dub. ... ... ... | "Kilronan," Anglesea Road, Dublin, Ireland. |
| *Britton, R. B., M.B., B.S. Lond. ... ... ... | 33a Whiteladies Road, Clifton. |
| *Brocklehurst, R. J., M.A., D.M. Oxon. ... | Bristol University. |
| *Buckmaster, G. A., M.A., M.D. Oxon. ... ... | 6 Victoria Square, Clifton. |
| *Burdon-Cooper, J., M.D., B.Sc. Durh. ... ... | 12 The Circus, Bath. |
| *Burges, R. ... ... ... ... ... ... ... ... ... | Druid's Mead, Stoke Bishop, Bristol. |
| *Burges, C. R., M.B., Ch.B. ... ... ... ... | Ham Green Hospital, Pill, Nr. Bristol. |
| *Burgess, N. F. C., M.D. Cantab.... ... ... ... | Hereford House, Clifton. |
| *Burnett, R. H., M.B., B.S. Durh. ... ... ... | 479 Bath Road, Brislington, Bristol. |
| *Bush, G. B., M.B., Ch.B. Brist. ... ... ... | 5 Lansdown Place, Clifton. |
| | |
| *Cairns, F. J. ... ... ... ... ... ... ... ... | Devon House, Whitehall Road, Bristol. |
| *Carey, R. S., *O.B.E.*, B.A. Cantab. ... ... | 502 Bath Road, Brislington, Bristol. |
| *Carleton, H. H., M.A., M.D., B.Ch. Oxon. ... | 1 Lansdown Road, Clifton. |
| *Carwardine, Thomas, M.B., M.S. Lond.... ... | 16 Victoria Square, Clifton. |
| *Casson, E., M.D., Ch.B. Brist. ... ... ... ... | Dorset House, Clifton Down, Bristol. |
| *Cates, H. J., M.D. Lond. ... ... ... ... ... | Northwoods House, Winterbourne, Bristol. |
| *Cave, Edward J., M.D. Lond. ... ... ... ... | 16 The Circus, Bath. |
| *Chambers, E. R. ... ... ... ... ... ... ... | 9 Clifton Park, Clifton. |
| *Charles, J. R., M.A., M.D. Cantab.... ... ... | 5 Rodney Place, Clifton. |
| *Chesterman, J. T., M.D., B.S. ... ... ... ... | Glen Avon, Broomfield Road, Bath. |
| *Chitty, H., M.S. Lond. ... ... ... ... ... | 46 Pembroke Road, Clifton. |
| *Christofferson, P. E., M.B., Ch.B. Brist. ... | 8 Lansdown Place, Clifton. |
| *Clarke, R. C., *O.B.E.*, M.B., Ch.B. Brist. ... | 29 Victoria Square, Clifton. |
| *Coates, Vincent M., *M.C.*, M.A., M.D., B.C. Cantab. ... ... ... ... ... ... ... | 10 The Circus, Bath. |
| Connellan, P. S. ... ... ... ... ... ... ... | 12 Bloomsbury Street, London, W.1. |
| *Cookson, R. G. ... ... ... ... ... ... ... | 5 Worcester Terrace, Clifton. |
| *Coombs, Carey, M.D. Lond. ... ... ... ... | 3 Pembroke Road, Clifton. |
| *Cooper, William Russell ... ... ... ... ... | 13 Westfield Park, Redland, Bristol. |
| *Corfield, C., M.D. Durh. ... ... ... ... ... | 5 Kensington Place, Clifton. |
| *Cory, R. A. S.; M.B., Ch.B. Brist. ... ... ... | Montego Bay, Jamaica, B.W.I. |
| *Cossham, W. L., M.B., Ch.B. Brist. ... ... | 3 Claremont Road, Bishopston. |
| *Courtney, R. M., M.B., Ch.B. Glasg. ... ... | 487 Gloucester Road, Bristol. |
| *Coxon, Beatrice .. ... ... ... ... ... ... | 16 Richmond Hill, Clifton. |
| Cridland, A. B. ... ... ... ... ... ... ... | Salisbury House, Chapel Ash, Wolverhampton. |
| *Crossman, F. W., M.B. Durh. ... ... ... ... | White's Hill, Hambrook, near Bristol. |
| *Collinson, E. M. D. M., M.B., Ch.B. Brist. ... | 15 All Saints Road, Clifton. |
| *Cross, Clara D., M.D. ... ... ... ... ... | 17 Midford Road, Odd Down, Bath. |
| | |
| *Dacre, John ... ... ... ... ... ... ... ... | 14 Eaton Crescent, Clifton. |
| *Datta, S. S. M.D. Brist. ... ... ... ... ... | Snowdon House, Fishponds, Bristol. |
| *Davies, E. D. D. ... ... ... ... ... ... ... | Standish House, Stonehouse, Glos. |
| Devine, H., *O.B.E.*, M.D. Lond. ... ... ... | Holloway Sanatorium, Virginia Water. |
| *Dawson, W. R., *O.B.E.*, M.D. Dub.... ... ... | 18 Brock Street, Bath. |
| *Dix, C. ... ... ... ... ... ... ... ... ... | 11 Victoria Square, Clifton. |
| *Dixon, Helen M., M.B., B.Ch. Brist. ... ... | 10 Whatley Road, Clifton. |
| *Dixon, T. B. ... ... ... ... ... ... ... ... | 11 Pembroke Road, Clifton, Bristol. |
| *Duerden, J. R., M.B., Ch.B. Brist. ... ... | 149 Bell Hill Road, St. George, Bristol. |
| *Dutton, Hilda R. ... ... ... ... ... ... | 405 Stapleton Road, Bristol. |
| *Dyer, Walter Percy ... ... ... ... ... ... | West View, Westbury-on-Trym, Bristol. |
| | |
| *Eglinton, J. H. C. ... ... ... ... ... ... | Street, Somerset. |
| *Elkington, G. W., M.B., B.S. Lond. ... ... | Malvern College, Malvern. |
| Elliot Bros. Ltd.... ... ... ... ... ... ... | 20-22 O'Connell Street, Sydney, N.S.W., Australia. |

Elliott, F. Percy, M.B., C.M. Aberd. ... ... 113 Grove Rd., Walthamstow, London, E.17.
*Elliott, W. H. A., M.B., B.S. Lond. ... ... 116 Pembroke Road, Clifton.

*Faill, C. J. C. ... ... ... ... ... ... ... ... 19 Portland Square, Bristol.
*Fallon, Col. J. ... ... ... ... ... ... ... ... 35 Henleaze Gardens, Henleaze, Bristol.
*Faraker, E. C. ... ... ... ... ... ... ... ... 85 Coldharbour Road, Westbury Park, Bristol.
*Farquharson, J. L. ... ... ... ... ... ... ... Breda, Weston-super-Mare.
Farnfield, W. W. ... ... ... ... ... ... ... Clive Vale, Gillingham, Dorset.
*Fawn, G. F. ... ... ... ... ... ... ... ... ... 6 Oakfield Road, Clifton.
*Fayle, B. W. D., M.B., Ch.B. ... ... ... ... Reine Barnes, Dursley, Glos.
*Fells, A., M.B., C.M. Edin. ... ... ... ... 6 Elton Road, Clifton.
*Fells, G. R., M.B., Ch.B. Brist. ... ... ... Eaton House, Southmead Road, Bristol.
*Fells, R. R., M.B., B.S. Lond. ... ... ... 12*d* Cotham Road, Bristol.
*Feneley, G. L., M.B., Ch.B. ... ... ... ... Englewood, Stoke Lane, Westbury-on-Trym.
*Firth, J. L., M.D., M.S. Lond. ... ... ... 8 Victoria Square, Clifton.
*Fisher, A. C., M.B., Ch.B. Brist. ... ... ... Kalene Hill, Mwini Lunga, N.W. Rhodesia.
*FitzGibbon, G. M., M.D. ... ... ... ... ... Bristol General Hospital.
*Flemming, A. A. G. ... ... ... ... ... ... ... Bristol Royal Infirmary.
*Flemming, A. L., M.B., Ch.B. Brist. ... ... 48 Pembroke Road, Clifton.
*Flemming, C. E. S. ... ... ... ... ... ... ... Manvers House, Bradford-on-Avon.
*Forrester-Brown, Maud, M.D., M.S. Lond. ... 22 Coombe Park, Bath.
*Foss, E. V., M.D., Ch.B. Brist. ... ... ... Summer Hill House, St. George, Bristol.
*Fox, F. E., B.A. Camb. ... ... ... ... ... ... Brislington House, Brislington, Bristol.
*Fraser, A. D., M.A., M.B., Ch.B. Glasg. ... 4 Alexandra Road, Clifton.
*Fraser, D., M.B., Ch.B. Edin. ... ... ... ... Westgate, Dursley, Glos.
*Freeth, H., M.D. Dub. ... ... ... ... ... ... 93 Redland Road, Bristol.
*Fuller, H. L. ... ... ... ... ... ... ... ... 9 Gay Street, Bath.

*Garden, R. R., M.A., M.B., B.Ch. Aberd. ... 9 Harley Place, Clifton.
Garland, E. B., M.B., C.M. Edin. ... ... ... 114*a* Hampton Road, Redland, Bristol.
*Gee, C. A. H., M.B., Ch.B. Brist. ... ... ... Portbury, Nr. Bristol.
*Giraldi, J. J., M.B., Ch.B. Brist. ... ... ... Gibraltar.
*Glenny, E. T., M.B., B.S. Lond. ... ... ... 102 Pembroke Road, Clifton.
*Gordon, R. G., M.D., B.Sc. Edin. ... ... ... 9 The Circus, Bath.
*Gorham, A. P., M.B., Ch.B. Brist. ... ... ... 53 Westbury Road, Westbury-on-Trym.
*Gornall, W. A., M.D., Brist. ... ... ... ... Orchard House, Nailsea, Somerset.
*Greenlaw, Madge E., M.B., Ch.B. Brist. ... 194 Redland Road, Bristol.
Griffiths, Cornelius A. ... ... ... ... ... ... 35 Newport Road, Cardiff.
*Griffiths, S. J. H., M.B., Ch.B. Brist. ... ... 1 Victoria Square, Clifton.
*Groves, E. W. Hey, M.D., M.S. Lond. ... ... 25 Victoria Square, Clifton.

*Hall, E. George, M.B. Lond. ... ... ... ... 42 Apsley Road, Clifton, Bristol.
*Hall, I. Walker, M.D., Ch.B. Vict. ... ... Shortwood Lodge, Pucklechurch, Glos.
*Ham, C. I., M.B., Ch.B. Brist. ... ... ... ... 16 Elm Lane, Redland, Bristol.
*Harris, H. Elwin, B.A., M.B. Cantab. ... ... 13 Lansdown Place, Clifton
*Harris, H. E., M.A., M.B., B.Ch. Cantab. ... 13 Lansdown Place, Clifton.
*Harsant, W. H. ... ... ... ... ... ... ... ... Tower House, Clifton Down Road, Clifton.
*Hartley, G., M.B., B.S Lond. ... ... ... ... 28 Pembroke Road, Clifton.
*Henderson, James, M.B., Ch.B. Aberd. ... ... The City Sanatorium, Yardley Road, Birmingham.
*Hector, F., M.D. Lond. ... ... ... ... ... ... Chelsea Hospital for Women, Arthur Street, Chelsea, S.W.3.
*Herapath, C. E. K., *M.C.*, M.D. Lond.... ... Ormlie, Clifton.
*Heron, Gordon, M.B., Ch.B. Brist. ... ... ... 34 Gloucester Road, Bristol.
*Heron, Gordon, Mrs., M.B., Ch.B. Brist. ... 34 Gloucester Road, Bristol.
*Hooker, J. A., M.B., Ch.B. Brist. ... ... ... 16 St. Edyth's Road, Sea Mills.
*Hosken, J. G. F. ... ... ... ... ... ... ... ... Uley, Glos.
Hospital, Bristol General (*Secretary*, T. W. Gregg).
*Houghton, Lydia M., M.B., B.S. Lond. ... ... 74 Church Road, Redfield, Bristol.

| | |
|---|---|
| *Hughes, T. I. ... ... ... ... ... ... ... | Bristol General Hospital. |
| *Hughes F. W. Terrell, M.B., Ch.B. Brist. ... | Chew Magna, Somerset. |
| *Hunter, F. S. ... ... ... ... ... ... ... ... | c/o Messrs. J. Wright & Sons Ltd. Publishers, Bristol. |
| *Iles, A. E., *O.B.E.*, M.B., B.S. Lond. ... ... | 17 Victoria Square, Clifton. |
| Infirmary, Bristol Royal (*Secretary*, E. C. Smith). | |
| Ingle, C. Durban ... ... ... ... ... ... ... | Somerton, Somerset. |
| *Irving, J. T., M.A., M.D., Ph.D. ... ... ... | 5 The Glen, Durdham Down, Bristol. |
| *Jackman, W. A., M.B., Ch.B. Brist. ... ... ... | 15 Mortimer Road, Clifton. |
| *James, J. A., M.D. Lond. ... ... ... ... ... | 5 Rodney Place, Clifton. |
| *James, April D., M.B., Ch.B. Brist. ... ... | Queen's Hospital, Birmingham. |
| *Jenkins, R. D. ... ... ... ... ... ... ... ... | Almondsbury. |
| *Jenkins, F. G., M.B., Ch.B. Brist. ... ... ... | 51 Redcliff Hill, Bristol. |
| *Johnson, R. G., M.B. Lond. ... ... ... ... | 119 Redland Road, Bristol. |
| *Joll, C. A., M.B., M.S. Lond. ... ... ... ... | 64 Harley Street, London. |
| *Joscelyne, P. C., M.B., Ch.B. Brist.... ... ... | 70 Longmead Avenue, Bishopston, Bristol. |
| *Kemm, N. F., M.B., B.S. Lond. ... ... ... | 188 Redland Road, Bristol. |
| *Kennedy, W. P., M.D. Dub. ... ... ... ... | 3 Gay Street, Bath. |
| *Kerfoot, Stanley J. ... ... ... ... ... ... ... | 194 Redland Road, Bristol. |
| *Kingston, S. H., M.B., Ch.B. Brist. ... ... | 3 Whiteladies Road, Clifton. |
| *Kyle, H. G., M.A., M.D., B.Ch. Oxon. ... ... | 31 Westbury Road, Bristol. |
| *Lace, F. ... ... ... ... ... ... ... ... ... ... | 5 Gay Street, Bath. |
| *Langley, G. F., M.B., Ch.B. Brist. ... ... ... | Royal Free Hospital, London, W.C. |
| *Lees, E. Leonard, M.D., C.M. Edin. ... ... | 22 Richmond Hill, Clifton. |
| *Legat, R. Eddowes, M.B., C.M. Edin. ... ... | Murray House, Staple Hill, Bristol. |
| Leigh, W. W. ... ... ... ... ... ... ... ... | Llansamnor House, Cowbridge, Glam. |
| *Leonard, R. C., M.D. Durh. ... ... ... ... | Bower Ashton, Bristol. |
| *Lester, Muriel A., M.B., B.S. ... ... ... | 110 Avon Vale Road, Redfield, Bristol. |
| *Levis, J. S., *M.C.*, M.B., B.Ch., N.U.I. ... ... | 20 Gay Street, Bath. |
| *Linton, Marion S., M.B. Lond. ... ... ... | 21 Oakfield Road, Clifton. |
| *Lister, A. E. J., M.B., B.S. Lond. ... ... ... | 86 Pembroke Road, Clifton. |
| *Lister, L. Margaret ... ... ... ... ... ... ... | 12 All Saints Road, Clifton. |
| *Logan, H. B. ... ... ... ... ... ... ... ... ... | Elmwood, Aberdeen Road, Clifton. |
| *Lucas, R. P., M.B., Ch.B. Brist. ... .. ... | Bristol Royal Infirmary. |
| *Lucas, J. E. ... ... ... ... ... ... ... ... ... | 48 Coronation Road, Bristol. |
| *Lucas, J. J. S., M.D. Lond. ... ... ... ... | 186 Wells Road, Totterdown, Bristol. |
| *Lysaght, A. C. ... ... ... ... ... ... ... ... | 8 Clifton Hill, Bristol. |
| *Macfie, J. D., M.B., Ch.B. Glasg. ... ... ... | Winsley Sanatorium, near Bath. |
| Mackinnon, Charles, M.B., C.M., M.A. Glasg. | Davaar, Cirencester. |
| *MacLachlan, Alexander Macalister, M.B., Ch.B., Glasg. ... ... ... ... | 65 Redcliff Hill, Bristol. |
| Maclean, Sir Ewen J., M.D., C.M. Edin. ... | 12 Park Place, Cardiff. |
| *MacLeod, Donald, M.B., C.M. Edin. ... ... | 84 Redland Road, Redland, Bristol. |
| *MacLeod, G., *M.C.*, M.D., Ch.B. ... ... ... | "Wargrave," Clevedon. |
| *MacWatters, J. C., *M.B.E.* ... ... ... ... | Almondsbury, near Bristol. |
| Marle, S. ... ... ... ... ... ... ... ... ... | Newbridge Road, Bath. |
| *Marshall, A. H., M.B., Ch.B. Bristol. ... ... | Odelos, Canford Lane, Bristol. |
| Marshall, Arthur L., M.B., B.A. Cantab. ... | Raveningham, Norwich. |
| *Martin, P. S. ... ... .. ... ... ... ... | Victoria Quadrant, Weston-super-Mare. |
| Mather, J. S., M.B., C.M. Aberd. ... ... ... | Lawrence Hill House, Bristol. |
| *Mayes, F. J. A. ... ... ... ... ... ... ... ... | 79 Pembroke Road, Clifton. |
| *McCrea, Phillip, M.D., B.Ch. Dub. ... ... ... | 56 Kellaway Avenue, Bristol. |
| *McDowell, Andrew, M.B., Ch.B. Edin. . ... | The General Hospital, Bristol. |
| *McKenzie, W. G., *M.C.* ... ... ... ... ... | Ministry of Health Regional Med. Office, 3 Victoria Road, Darlington, Co., Durham |
| *McLannahan, J. G. ... ... ... ... ... ... | The Mount, Stonehouse. |
| *Meadows, G. ... ... ... ... ... ... ... ... ... | Auteuil, Southmead Road, Bristol. |
| Miall, C. L'O. ... ... ... ... ... ... ... ... | Elm Hayes, Paulton, near Bristol. |
| *Milling, T., M.B., Ch.B. Belfast ... ... ... | 1 Vicarage Road, Southville, Bristol. |

| | |
|---|---|
| *Milner, A., M.B., B.Ch. Dub. ... ... ... | 61 Cotham Brow, Bristol. |
| *Minchin, R. L. Haviland ... ... ... ... | 2nd County Mental Hospital, Coney Hill, Barnwood, Gloucester. |
| *Moore, R. M., M.A., M.B., Ch.B. Cantab. ... | Mundens, Malmesbury, Wilts. |
| *Moore, C. A., M.B., M.S. Lond. ... ... ... | 56 St. Paul's Road, Clifton. |
| *Moore, L. A.... ... ... ... ... ... ... ... | Hillsborough, Cotham Park, Bristol. |
| *Morris, L. N., M.B., Ch.B. Brist. ... ... ... | 24 All Hallows Rd., Upper Easton, Bristol |
| *Morison, A. G., M.A., M.B., Ch.B., D.P.H. ... | 34 Woodstock Road, Redland, Bristol. |
| *Mundy, G. S., M.B., B.Ch. Brist. ... ... ... | Homewood, Coombe Dingle, Bristol. |
| *Murphy, F. D., M.B., Ch.B., B.Sc. Cork ... | Queen Mary's Hospital, Stratford, London, E. |
| *Myles, G. T. ... ... ... ... ... ... ... ... | 7 Apsley Road, Clifton. |
| *Myles, Q. St. L., M.A., B.M., B.Ch. Oxon. | 7 Apsley Road, Clifton. |
| *Neild, Newman, M.B., B.Ch. Vict. ... ... ... | 9 Richmond Hill, Clifton. |
| *Newnham, W. H. C., M.B., M.A. Cantab. ... | 3 Lansdown Place, Victoria Square, Clifton. |
| *Nixon, J. A., *C.M.G.*, M.D. Cantab. ... ... | 7 Lansdown Place, Clifton. |
| *Noble, J. Innes, M.B., Ch.B. Liverp. ... ... | 102 Pembroke Road, Clifton. |
| *Nott, Winifred, M.B., Ch.B. Brist. ... ... ... | "Fassiefern," Upper Cranbrook Road, Redland, Bristol. |
| *O'Brien, C., M.B., Ch.B. ... ... ... ... ... | Bristol Children's Hospital. |
| *O'Donohue, A., M.B., Ch.B. ... ... ... ... | The Children's Hospital, Bristol. |
| *Ormerod, G. L., M.A., M.B., B.Ch. Cantab. ... | 2 Henleaze Road. |
| *Ormerod, H. Lawrence, M.D., B.Ch. R.U.I. ... | 2 Henleaze Road, Westbury-on-Trym, Bristol. |
| Orr-Ewing, H. J., *M.C.*, M.D. Lond. ... ... | English Mission Hospital, Jerusalem. |
| *Parker, George, M.A., M.D. Cantab. ... ... | 9 Pembroke Road, Clifton. |
| *Parkes, J. A., M.B., Ch.B. Liverp. ... ... ... | 8 South Road, Redland, Bristol. |
| Parkinson, Lt.-Col. G. S., *D.S.O.*, R.A.M.C. ... | R.A.M.C. Mess, Grosvenor Place, London, S.W.1. |
| *Parry, R. H., M.B., B.S. Lond. ... ... ... | Newby, Holmes Grove Road, Henleaze. |
| Parsons, Sir J. H., M.B., B.S., B.Sc. Lond. ... | 54 Queen Anne Street, London, W. |
| *Paul, R. G. ... ... ... ... ... ... ... ... | Bristol Royal Infirmary. |
| *Pearce, J. L., M.B., Ch.B. Edin. ... ... ... | Hambrook Court, near Bristol. |
| *Perry, Bruce, M.B., Ch.B. Brist. ... ... ... | 4 Richmond Park Road. |
| *Phillips, P., M.B., Ch.B. Brist. ... ... ... ... | Southmead Hospital, Bristol. |
| *Pinkerton, J. M., B.Sc. Wales; M.B., Ch.B. Glasg. | 5 The Circus, Bath. |
| *Pollard, J., M.B., B.Ch., B.A.O., N.U.I. ... | 88 Coronation Road, Bristol. |
| *Potter, Mabel F., M.B., Ch.B. ... ... ... | 1 Mortimer Road, Clifton. |
| *Powell, H. F., M.D. Brux. ... ... ... ... | Ellenborough Park, Weston-super-Mare. |
| *Powell, J. J., M.D. Lond. ... ... ... ... | 2 Gloucester Road, Bishopston, Bristol. |
| *Price, A., M.B., Ch.B. Brist. ... ... ... ... | 16 Cavendish Road, Henleaze, Bristol. |
| *Price, N. L., M.B., Ch.B. Brist. ... ... ... | General Infirmary, Salisbury, Wilts. |
| Pridie, K. H., M.B., B.S. Lond. ... ... ... ... | Frenchay Orthopædic Hospital, Bristol. |
| *Pring, C. E. ... ... ... ... ... ... ... ... | 14 West Park, Clifton, Bristol. |
| *Prowse, D. C., M.B., Ch.B. Brist. ... ... | Wigmore House, Thornbury, nr. Bristol. |
| *Rayner, D. C., Ch.M. Brist. ... ... ... ... | 9 Lansdown Place, Clifton, Bristol. |
| *Redman, Ethel, M.B., Ch.B. ... ... ... ... | Winford Orthopædic Hospital, nr. Bristol. |
| *Renton, R. A. S., *M.C.*, M.D. Durh. ... ... | Emsworth House, Clevedon. |
| *Robertson, D., M.B., Ch.B. Edin. ... ... ... | 205 Wells Road, Knowle, Bristol. |
| *Rogers, Herbert, M.B., Ch.B. Brist. ... ... | 11 Clifton Hill, Bristol. |
| *Rolfe, R., B.A., M.B., B.C. Cantab. ... ... | Park House, St. Andrew's Road, Avonmouth. |
| *Ross, C. C., M.D. Man.... ... ... ... ... ... | Minnedosa, Manitoba, Canada. |
| *Rowley, A. T. ... ... ... ... ... ... ... | Wrington. |
| *Rudolf, G. R. A. de M. ... ... ... ... ... | Brentry Colony, Westbury-on-Trym, Bristol. |
| *Salisbury, Walter, M.D., M.S. Lond. ... ... | Woodlands, 20 Billing Road, Northampton. |
| Savage, W. G., M.D., B.Sc. Lond. ... ... ... | "Bourn," 7 Trewartha Park, Weston-super-Mare. |

*Saxby, Ida, D.Sc., Lond. ... ... ... ... ... ... 24 Durdham Park, Bristol.
*Scarff, G., M.B., Ch.B. Edin. ... ... ... ... 5 Rodney Place, Clifton.
*Scott, H. H. ... ... ... ... ... ... ... ... ... 78 Old Market Street, Bristol.
*Shaw, J. E., M.B., C.M. Edin. ... ... ... ... 23 Caledonia Place, Clifton.
*Shepherd, H. L., M.B., Ch.M. Brist. ... ... 16 Victoria Square, Clifton.
*Short, A. R., B.Sc., M.D., B.S. Lond. ... ... 69 Pembroke Road, Clifton.
*Smith, G. Cockburn, M.D. Brux. ... ... ... 12 South Road, Newton Abbot.
*Smith, T. Wilson, M.D. Lond. ... ... ... ... 26 The Circus, Bath.
*Smythe, H. J. D., *M.C.*, M.B., M.S. Lond. ... 15 Eaton Crescent, Clifton.
*Somers, Mary F. E. ... ... ... ... ... ... ... 2 Ashcombe Road, Weston-super-Mare.
*Spoor, A., M.B., B.Ch. Cantab. ... ... ... The Hydro, Bristol.
*Staniland, M. F. ... ... ... ... ... ... ... ... 255 Stapleton Road, Bristol.
*Statham, R. S. S., *O.B.E.*, M.D., M.Ch. Brist. 10 Beaufort Road, Clifton, Bristol.
*Stewart, A. L., D.M., D.Ch. Brux. ... ... 31 Howard Road, Southville, Bristol.
*Stock, W. S. V., M.B. Lond. ... ... ... ... 1 Mortimer Road, Clifton.
*Stone, D. M., M.D., B.S. Lond. ... ... ... 28 Pembroke Road, Clifton, Bristol.
*Strover, H. W. M., *O.B.E.*, M.B., Ch.B. Aberd. ... ... ... ... ... ... ... ... 24 Gloucester Road, Bishopston, Bristol.
*Sutton, G. E. F., M.D. Lond. ... ... ... ... 1 Pembroke Road, Clifton.
*Symes, J. O., M.D. Lond. ... ... ... ... 6 Pembroke Vale, Clifton.

*Tasker, D. G. C., M.B., M.S. Lond. ... ... 10 Christchurch Road, Clifton.
*Taylor, A. L., M.D. Lond. ... ... ... ... ... Bristol General Hospital.
Taylor, A. L. ... ... ... ... ... ... ... ... ... Bristol Royal Infirmary.
*Taylor, H. N., M.A. Cantab., M.D. Dub. ... Hillside, Ebbw Vale.
*Temple, G. H., M.B., C.M. Edin. ... ... ... Ailanthus, Weston-super-Mare.
*Terry, C. H., M.A., M.B., B.Ch. Oxon. ... ... 15 The Circus, Bath.
Thin, James ... ... ... ... ... ... ... ... ... 54 & 55 South Bridge, Edinburgh.
*Thompson, J., M.B., Ch.B. Edin. ... ... ... Eastbourne House, Devizes.
*Thomas, J. D., M.B. Cantab. ... ... ... ... Dan-y-Graig, Cheyne Road, Stoke Bishop
*Todd, A. T., *O.B.E.*, M.B., Ch.B. Brist. ... ... 10 Tyndall's Park Road, Clifton.
*Tripp, Dorothy, M.H., M.B., Ch.B. Brist. ... Wesleyan Mission Hospital, Karim Nagar, H.E.H. The Nizam's Dominions, India.
*Trist, J. R. R., *M.C.* ... ... ... ... ... 120 Henleaze Road, Henleaze.
*Tryon, Victoria S., M.B., Ch.B. Brist. ... ... 3 Harley Place, Clifton.

*Walbank, A., M.B., Ch.B. Leeds ... ... ... 30 Whiteladies Road, Clifton.
*Walker, Cyril H., B.A., M.B. Cantab. ... ... 8 Oakfield Road, Clifton.
Wallace, S., O.B.E. ... ... ... ... ... ... ... 10 Knightstone Rd., Weston-super-Mare.
*Walsh, M. J., M.B., Ch.B. Dub. ... ... ... ... 2 Knowle Road, Bristol.
*Walters, C. F. ... ... ... ... ... ... ... ... 5 Mortimer Road, Clifton.
*Wansborough, T. B., M.B., Ch.B. Brist. ... ... 8 Mortimer Road, Clifton.
*Wanklyn-James, C. W., *O.B.E.* ... ... ... 3 Mortimer Road, Clifton.
*Warren, R., M.A., M.D. Oxon. ... ... ... ... Ellenborough Hall, Weston-super-Mare.
*Waterhouse, R., M.D. Lond. ... ... ... ... 3 The Circus, Bath.
*Watson-Williams, E., *M.C.*, B.A., M.B., Ch.M. Cantab. ... ... ... ... ... ... ... ... 12 Victoria Square, Clifton.
*Weddell, C. W. ... ... ... ... ... ... ... ... Boydville, 46 Miller St., West Melbourne, Australia
Weston, B. A., M.B. ... ... ... ... ... ... ... Brookside, Admaston, Wellington, Shropshire.
*Whelton, A., B.A., M.B., Ch.B. Cork ... ... Innisfree, Western Road, Cork.
*White, E. B. C. ... ... ... ... ... ... ... ... Mental Hospital, Fishponds, Bristol.
Whitehouse, H. Beckwith, M.S. Lond. ... 62 Hagley Road, Edgbaston, Birmingham.
*Wigoder, Sylvia Beatrice, M.A., M.D., Ph.D. Radium Centre, Bristol Royal Infirmary.
Willcox, R. Lewis ... ... ... ... ... ... ... 97 London Road, Salisbury.
Williams, S. R., M.B. Lond. ... ... ... ... Buckfastleigh, Devon.
*Williams, A. R., M.B., Ch.B. Brist. ... ... 1 Horsley Hill Road, South Shields.
*Wills, W. K., *O.B.E.*, M.B., B.C. Cantab. ... 1 Victoria Square, Clifton.
*Wood Duncan ... ... ... ... ... ... ... ... 5 Eaton Crescent, Clifton.
*Wood, W. V., *M.C.*, B.A. Cantab. ... ... ... Henley Lodge, Yatton, Som.
*Wright, A. J. M.B., B.S. Lond. ... ... ... Clifton Park, Clifton.

*Zealand, L., M.D. Man. ... ... ... ... ... Brecon Lodge, Westbury-on-Trym, Bristol.

### Publications Received

From Baillière, Tindall and Cox:

*Research Work on the Pneumococci and their Enzymes and its Significance in Lobar Pneumonia.* By A. Cowan Guthrie, M.B. Price 7s. 6d.
*Physicians' Manual of Birth Control.* By Antoinette F. Konikow, M.D. Price 12s. 6d.

From John Bale, Sons and Danielsson Ltd.:

*Diseases and Disorders of the Digestive Organs.* By Adolphe Abrahams, O.B.E., M.D., F.R.C.P. Price 2s. 6d.
*The Acute Abdomen.* By C. H. Fagge, M.S., F.R.C.S. Price 2s. 6d.
*Radium and Cancer.* By H. S. Souttar, C.B.E., M.D., M.Ch., F.R.C.S. Price 2s. 6d.
*Acute Otitis Media.* By W. M. Mollison, C.B.E., M.A., M.Ch., F.R.C.S. Price 2s. 6d.
*Criminal Abortion.* By L. A. Parry, M.D., B.S., F.R.C.S.

From William Heinemann (Medical Books) Ltd.:

*Textbook of Gynæcology.* By Sidney Forsdike, M.D., B.S., F.R.C.S. Price 15s.
*Hydrotherapy and Physiotherapy.* By L. C. E. Calthrop, M.B., M.R.C.S., L.R.C.P. Price 5s.
*Science of Signs and Symptoms.* By R. J. S. McDowall, D.Sc., M.B., F.R.C.P. Price 21s.
*Specific Changes in the Blood Serum.* By S. G. T. Bendien. Price 10s. 6d.
*Hygiene of Marriage.* 3rd Ed. By I. E. Hutton, M.D. Price 5s.

From His Majesty's Stationery Office:

*The Intervertebral Discs* (Medical Research Council Special Report, No. 161). By Ormond A. Beadle. Price 2s.

From The Lancet Ltd.:

*The Lancet Commission on Nursing.* Final Report. Price 2s. 6d.
*Guy's Hospital Reports.* October, 1931. Vol. 81, No. 4. Edited by A. F. Hurst, M.D. Price 12s. 6d.

From H. K. Lewis & Co. Ltd.:

*Chest Disease in General Practice.* By Philip Elman, M.D., M.R.C.P. Price 15s.
*Heart and Spleen in Health and Disease.* By G. A. Stephens, M.D., B.S., B.Sc. Price 7s. 6d.

From Oxford University Press:

*Wayfaring in a Splint.* By O. Wallace. Price 2s. 6d.
*Diagnosis in Joint Disease.* By N. Allison, M.D., F.A.C.S., and R. K. Ghormley, M.D. Price 52s. 6d.
*Textbook of Massage and Remedial Gymnastics.* 3rd Ed. By L. L. Despard. Price 22s. 6d.

From Kegan Paul, Trench, Treubner & Co. Ltd.:

*Individual Sexual Problems.* By F. G. Crookshank, M.D., F.R.C.P. Price 2s. 6d.

From John Wright & Sons Ltd.:

*The British Journal of Surgery.* Vol. XIX., No. 75. January, 1932. Price 12s. 6d.
*Synopsis of Midwifery and Gynæcology.* By A. W. Bourne, F.R.C.S. Price 15s.
*An Index of Prognosis and End-Results of Treatment.* 4th Ed. By Various Writers. Edited by A. Rendle Short, M.D., B.S., B.Sc., F.R.C.S. Price £2 2s.
*A Theory of the Formation of Animals.* By W. T. Hillier, M.R.C.S., L.R.C.P. Price 8s. 6d.

